# Costs of sickness absence in a service company in Colombia: case analysis by gender and occupation

**DOI:** 10.47626/1679-4435-2022-757

**Published:** 2023-02-03

**Authors:** Francisco Palencia-Sánchez, Martha Isabel Riaño-Casallas

**Affiliations:** 1 Facultad de Medicina, Pontificia Universidad Javeriana, Bogotá, Bogotá, Colombia; 2 Facultad de Ciencias Económicas, Universidad Nacional de Colombia, Bogotá, Bogotá, Colombia

**Keywords:** sick leave, absenteeism, cost, sickness absence patterns, chronic diseases., licença médica, absenteísmo, custos, padrões de licença médica, doenças crônicas.

## Abstract

**Introduction:**

Sickness absence is a phenomenon that has an impact on productivity, costs, and the working environment.

**Objectives:**

To understand the patterns of sickness absence by gender, age, and occupation, as well as its association with cost in a service company.

**Methods:**

We conducted a cross-sectional study based on the sick leave data for 889 employees in one service company. The total number of sick leave notifications registered was 156. We performed a t-test for gender and a non-parametric test for the mean differences related to cost.

**Results:**

We found that women registered more sick days than men, accounting for 68.59% of all sick days recorded. Absence due to sickness was more common in the age range of 35-50 years for both men and women. The mean number of days lost was 6, and the average cost was 313 U.S. dollars. Chronic disease was the main cause of sick leave, representing 66.02% of all absent days. There were no differences in the mean number of days of sick leave between men and women.

**Conclusions:**

There is no statistical difference in the number of days of sick leave between men and women. The costs of absence related to chronic disease are higher than those for other causes, so it is good practice to try developing health promotion programs in the workplace to prevent chronic disease in the working age population and reduce its associated costs.

## Abstract (pt)

**Introdução:**

A licença médica é um fenômeno que afeta a produtividade, os custos e o ambiente de trabalho.

**Objetivos:**

O objetivo deste estudo é compreender os padrões de licença médica por sexo, idade, ocupação e sua relação com os custos de uma empresa de serviços.

**Métodos:**

Realizamos um estudo transversal com base nos dados de licença médica de 889 funcionários de uma empresa de serviços. Registraram-se 156 notificações de licença médica. Realizamos um teste *t* por sexo e um teste não paramétrico para testar as diferenças médias relacionadas aos custos.

**Resultados:**

Verificamos que as mulheres registraram mais dias de licença médica (68,59%) do que os homens. A licença médica foi mais comum na faixa etária de 35-50 anos, tanto para homens quanto para mulheres. O número médio de faltas foi de 6 dias e os custos médios foram de 313 dólares americanos. As doenças crônicas foram a principal causa de licença médica (66,02%). Não houve diferença da média de dias de licença médica entre homens e mulheres.

**Conclusões:**

Não houve diferença estatística de dias de licença médica entre homens e mulheres. Os custos de licença médica relacionados a doenças crônicas são mais altos do que os de outras causas, portanto constitui boa prática tentar desenvolver programas de promoção da saúde no ambiente de trabalho, para prevenir doenças crônicas na população economicamente ativa e reduzir seus custos associados.

## INTRODUCTION

One of the greatest problems faced by managers every day is how to deal with workers’ absence. This is because when one worker misses a day of work, many problems emerge. Clients do not receive their products, coworkers have an additional workload, managers should start looking for a replacement, training is required, and accidents could even occur because new employees start performing an unfamiliar task, and all these contribute to costs for the company.

In many studies, the focus on the cost of sick leave is on healthcare services, or a clinical perspective is taken; for example, we found studies covering the cost of hip replacements,^^1^^ Parkinson’s disease,^^2^^ elective surgery,^^3^^ treatment for low back pain,^^4^^ Ménière’s disease,^^5^^ arthritis,^^6^^ and depression, among others. This is because sickness absence is a widely studied integrated measure of health status.^^7^^ Nevertheless, employers must know the economic consequences of the sick leave taken by their employees, and the main diseases from which their employees suffer, to start to design interventions to reduce the prevalence of those diseases and promote health in the workplace. Back pain, stress, depression, and anxiety cause 10% of sickness-absence days,^^8^^ and mental health problems, in particular, apparently drive a pattern of increasing sickness absence over time.^^7^,^9^^

Absence is defined as the number of days in which a worker is absent from work, while sickness absence is defined by MacGregor & Cunningham^^10^^ as absence from work because of an adverse health condition. Presenteeism refers to reduced productivity while a sick worker is working (and being paid).^^11^^ For example, a systematic review of the economic evaluation of workplace health promotion programs in Europe shows that most studies have, as their primary outcome, the lost or gained productivity expressed in reduced numbers of sick leave days or absence.^^12^^ Other studies try to estimate the effectiveness of programs to reduce sickness absence.^^8^,^9^,^13^,^14^^

Additionally, in most cases, the measurement of absence is based on a single question and is self-reported.^^10^,^15^^ In a study of two public employers, the mean number of days of sickness absence was 4.27 per year^^10^^ and, in a study in Norway, the average number of days of absence financed by public funds was 4.3 between 1996 and 1998, and 5.5 between 2003 and 2005,^^16^^ but, when there was a performance pay scheme, the sickness absence rates were 4.6 and 5.0 for 2003 and 2005 respectively, and changed to 5.0 and 6.0 without performance pay. In relation to gender differences, according to the Swedish Social Insurance Agency, women have more days of sick leave than men, mostly because of psychological problems such as stress and depression.^^15^^ In the case of age-related differences, there have been no conclusive findings.^^17^^

The costs of absence are distinctly higher than the direct medical costs,^^12^^ but some authors affirm that using wages to evaluate the cost of absence will underestimate that cost,^^18^^ because sometimes absence does not change production levels.^^19^^ The Chartered Institute for Personnel and Development estimated that in 2009 the cost of sickness absence to employers was exceeding £90 per day per employee.^^8^^ The costs of sickness absences constitute a heavy burden on private businesses, and these costs have not diminished over time.^^16^^ The costs include not only the salary of the absent employee, but also payments for overtime work and replacement workers, as well as management costs.

There is a substantial variation in sickness absences across firms, but firms can reduce absences by implementing broad programs, including performance pay, general improvements in working conditions, and programs to strengthen workers’ loyalty to the firm.^^20^^ To implement such programs, it is first necessary for companies to have data about the causes and cost of absence, to help design programs that reduce the consequences of absence from work due to illness. Such programs could be health promotion or return to work programs. The workplace integration of health protection and health promotion activities is becoming a new standard for safeguarding the health and safety of the workforce.^^21^^

Companies are worried about increases in sickness absence. Absence due to sickness is a problem that companies must deal with. However, there are not enough studies on the costs of this phenomenon for companies and workers. Knowing the magnitude of the phenomenon can allow companies to design strategies to control the situation, since absence related to medical disabilities can be an indicator of the presence of occupational or work-related illnesses. For this reason, the aim of this article is to study the patterns of sick leave and its cost by gender, type of occupation, and cause of sick leave in a service company in Colombia as a case analysis.

## METHODS

A cross-sectional analysis was conducted using secondary data. The data were collected from the absenteeism database of a service company in Colombia for 2015. This company has 889 employees in different locations in the country. The unit of analysis was the sick leave register provided by the medical doctor of the health insurance company, for each worker. The total number of periods of sick leave registered for this period was 156. The database provided by the company was completely anonymized. The variables included were:

Occupation: we classified the occupation according to the international Standard Occupational Classification of the International Labor Organization.^^22^^

Age: measured in years and grouped into three categories.

Number of sick leave days: days off from work spent at home for health reasons.

Sick leave payment by the firm: payment for period of sick leave of up to 2 days.

Sick leave payment by social security system: payment for period of sick leave of more than 2 days.

Short-term sick leave: sick leave of between 2 and 7 days.

Long-term sick leave: sick leave of 7 days or more.

Cause of sick leave: this is related to the impact on different organic systems. We used the International Statistical Classification of Disease and Related Health Problems, 10th revision (ICD-10).^^23^^

Cost of absence: the cost of a day’s labor. This information came from the payroll of the company. To calculate this cost, we considered that the employee was paid for the first two days of sick leave by the company and from the third day onward by health insurance according to the law in Colombia.^^24^^

Type of medical care: we classified the cause of the visit for medical care by inference from the sick leave register, using six categories - primary care (medical appointment during working hours); care for an infectious disease such as flu; care for a chronic disease, which covered a broader spectrum of causes; maternity care related to pregnancy complications; post-operative care; and trauma care.

Statistical analysis was performed using Stata version 14.0 (Stata Corporation LP, College Station, TX, USA). First, we performed a descriptive analysis by gender according to the variables included in the database and variables that we formed using the database. We conducted an analysis by gender and by other variables such as occupation, age, cause of temporary sick leave (according to the ICD-10),^^23^^ and period of sick leave. After this, we graphically tested the normality of the distributions of days of sick leave and cost between males and females. Then we tested the difference in means of the number of days of sick leave registered, the number of days of sick leave for each cause, and the total cost, by gender. We tested the normality of the distribution of days of sick leave and cost by occupation and type of medical care. If we did not observe a normal distribution, we performed the alternative test of a one-way analysis of variance (ANOVA) to test the mean difference between other groups.

We performed the Kruskal-Wallis H test, which is a rank-based nonparametric test than can be used to determine whether there are statistically significant differences between two or more groups with non-homogeneous variances and independent variables (continuous or ordinal). The Kruskal-Wallis test is a rank non-parametric alternative to the one-way ANOVA and an extension of the Mann-Whitney U test.^^25^^

The data for costs are expressed in U.S. dollars (USD) to allow international comparability.

## RESULTS

Table 1 shows that there were 107 periods of sick leave registered for women, with a mean of 6.5 days off work and a standard deviation (SD) of 9 days off work. For men, 49 periods of sick leave were registered, with a mean of 5 days off work and a SD of 6 days off work. The number of registered periods of sick leave paid for through the social security system was 53 for women, with a mean of 11 days off work and a SD of 11 days off work. For men, there were 24 registered periods of this kind, with a mean of 9 days off work and a SD of 7 days off work. Women and men in professional-type occupations were the most prevalent for absence in the period of analysis, followed by administrative technicians. Middle-aged women and men had the highest number of registered periods of sick leave. Most days of absence were taken by administrative technicians and professionals. Most days of sick leave were related to common diseases, followed by traffic accidents.

**Table 1:** Number of registered periods of sick leave by type of occupation, age, sick leave extension, type of medical care, and days of sick leave by type of occupation and cause

Variables	Male		Female	
	n	%	n	%
Number of registered periods by type of occupation				
Administrative technician	17	34.69	8	7.48
Managers	0	0.00	2	1.87
Professional	29	59.18	86	80.37
Qualified manual worker	0	0.00	10	9.35
Unqualified manual worker	3	6.12	1	0.93
Total	49	100.00	107	100.00
Number of registered periods by age (years)				
18-34	5	10.20	29	27.10
35-50	23	46.94	72	67.29
≥ 51	21	42.86	6	5.61
Total	49	100.00	107	100.00
Days of sick leave per type of occupation				
Administrative technician	143	55.86	29	4.20
Director	0	0.00	6	0.87
Professional	106	41.41	628	90.88
Qualified manual worker	0	0.00	25	3.62
Unqualified manual worker	7	2.73	3	0.43
Total	256	100.00	691	100.00
Extensions of registered sick leave periods				
Yes	5	10.20	11	10.28
No	44	89.80	96	89.72
Total	49	100.00	107	100.00
Cause of days of sick leave				
General disease	256	100.00	685	99.13
Injury by traffic accident	0	0.00	6	0.87
Number of periods of sick leave by type of medical care				
Primary care	1	2.04	1	0.93
Infectious disease care	20	40.82	55	51.40
Chronic disease care	21	42.86	31	28.97
Maternity care	0	0.00	13	12.15
Post-operative care	4	8.16	3	2.80
Trauma care	3	6.12	4	3.74
Total	49	100.00	107	100.00
Number of days of sick leave by type of medical care				
Primary care	1	0.39	1	0.14
Infectious disease care	45	17.58	119	17.22
Chronic disease care	169	66.02	405	58.61
Maternity care	0	0.00	126	18.23
Post-operative care	32	12.50	30	4.34
Trauma care	9	3.52	10	1.45
Total	256	100.00	691	100.00

Table 2 shows the distribution of sickness absence by gender and diagnosis. The main causes of sick leave were related to musculoskeletal disorders (MSD) in men, and to the respiratory system in women. The highest number of days of sick leave for administrative technicians (73% of such days) was related to MSD. For the next occupational category, managers, half of the days of sick leave were due to infection, and the other half to respiratory illness. The highest proportion of days of sick leave was due to cancer (48.91%) in the category of professionals; due to MSD (40%), for qualified manual workers; and, finally, due to respiratory disease (50%), for non-qualified manual workers.

**Table 2:** Number of registered periods and days of sick leave by gender and diagnosis

Variables	Male		Female	
	n	%	n	%
Number of registered periods for each ICD-10 chapter area				
Diseases of the circulatory system	0	0.00	1	0.93
Factors influencing health status and contact with health services	4	8.16	6	5.61
Diseases of the digestive system	3	6.12	4	3.74
Pregnancy, childbirth, and puerperium	0	0.00	10	9.35
Endocrine, nutritional, and metabolic diseases	0	0.00	1	0.93
Diseases of the genitourinary system	4	8.16	7	6.54
Symptoms, signs, and abnormal clinical and laboratory findings, not elsewhere classified	3	6.12	8	7.48
Certain infectious and parasitic diseases	3	6.12	20	18.69
Congenital malformations, deformations, and chromosomal abnormalities	1	2.04	0	0.00
Diseases of the nervous system	0	0.00	3	2.80
Diseases of the ear and mastoid process	0	0.00	1	0.93
Diseases of musculoskeletal system and connective tissue (no trauma)	17	34.69	7	6.54
Diseases of musculoskeletal system and connective tissue (trauma)	2	4.08	4	3.74
Diseases of the skin and subcutaneous tissue	1	2.04	1	0.93
Diseases of the respiratory system	8	16.33	22	20.56
Cancer	3	6.12	12	11.21
Total	49	100.00	107	100.00
Days of sick leave for each ICD-10-chapter area				
Diseases of the circulatory system	0	0.00	3	0.43
Factors influencing health status and contact with health services	23	8.98	74	10.71
Diseases of the digestive system	7	2.73	11	1.59
Pregnancy, childbirth, and puerperium	0	0.00	81	11.72
Endocrine, nutritional, and metabolic diseases	0	0.00	2	0.29
Diseases of the genitourinary system	18	7.03	15	2.17
Symptoms, signs, and abnormal clinical and laboratory findings, not elsewhere classified	3	1.17	13	1.88
Certain infectious and parasitic diseases	6	2.34	49	7.09
Congenital malformations, deformations, and chromosomal abnormalities	10	3.91	0	0.00
Diseases of the nervous system	0	0.00	6	0.87
Diseases of the ear and mastoid process	0	0.00	2	0.29
Diseases of the musculoskeletal system and connective tissue (no trauma)	133	51.95	24	3.47
Diseases of musculoskeletal system and connective tissue (trauma)	7	2.73	10	1.45
Diseases of the skin and subcutaneous tissue	2	0.78	2	0.29
Diseases of the respiratory system	20	7.81	52	7.53
Cancer	27	10.55	347	50.22
Total	256	100.00	691	100.00

ICD-10 = International Statistical Classification of Disease and Related Health Problems, 10th revision.

The highest proportion of days of sick leave per diagnosis for men (52%) was related to MSD, while half of sick leave days for women were related to cancer.

The cost of sick leave was on average 313 USD per registered period, and the total cost in the study period was 48,890 USD (Table 3). The responsibility for payment for the cost of absence fell mainly on health insurance (approximately 80%). More than half of the cost of absence was due to chronic diseases. The cost of absence by occupational type was higher for professionals, totaling 50.70% for men and 95.98% for women.

**Table 3:** Cost of sick leave, responsibility for payment, and cost per type of occupation and type of medical care

Variables	Male		Female	
	n	%	n	%
Registered periods of sick leave by payment (health insurance if period > 2 days, company if period ≤ 2 days)				
Company	25	31.65	24	31.17
Health insurance	54	68.35	53	68.83
Total	79	100.00	77	100.00
Days of sick leave by payment institution				
Company	43	16.80	91	13.17
Health insurance	213	83.20	600	86.83
Total	256	100.00	691	100.00
Cost of sick leave payment, by institution	USD		USD	
Company	2,544	17.61	3,900	11.32
Health insurance	11,899	82.39	30,546	88.68
Total	14,443	100.00	34,446	100.00
Cost of absence by occupational type				
Administrative technician	6,870	47.56	410	1.19
Director	0	0.00	495	1.44
Professional	7,468	51.70	33,067	95.98
Qualified manual worker	0	0.00	464	1.35
Unqualified manual worker	106	0.74	15	0.04
Total	14,445	100.00	34,446	100.00
Cost of sick leave by type of medical care				
Primary care (medical appointment)	39	0.27	89	0.26
Infectious disease care	2,611	18.08	5,363	15.57
Chronic disease care	9,705	67.19	21,734	63.10
Maternity care	0	0.00	5,946	17.26
Post-operative care	1,590	11.01	883	2.56
Trauma care	499	3.45	432	1.25
Total	14,444	100.00	34,446	100.00

USD = U.S. dollars.

**Figure 1:**
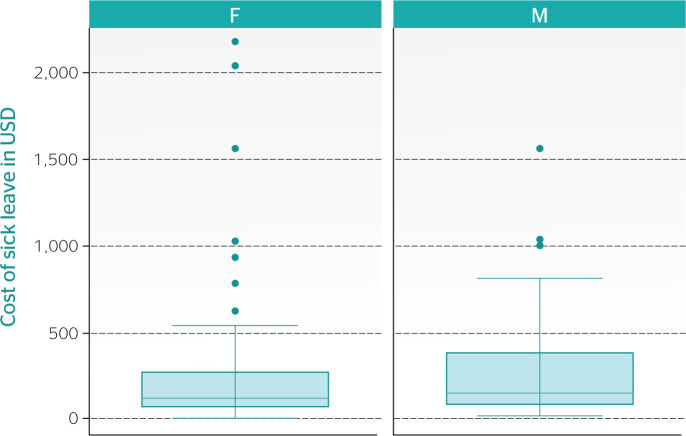
Cost of sick leave by gender. F = female: M = male; USD = U.S. dollars.

Figure 1 shows the distribution of the cost of sick leave by gender. There are more outliers related to the cost for women than for men, but the range between the 25th and the 75th percentiles is broader for men.

We performed Levene’s test for the homogeneity of variances in the days of sick leave between men and women, with the result that we could reject the equality of variances (p = 0.02630386). An independent t-test for unequal variances was run to determine whether there were differences in the periods of sick leave between female and male workers. The results showed that there was no statistically significant difference in the periods of sick leave between female workers (6.45 ± 0.86 days) and male workers (5.22 ± 0.82 days), t (154) = 1.0338, p = 0.3030 (based on a two-tailed significance level).

We then performed Levene’s test on the cost, with the result that we could not reject the equality of variances (p = 0.139). An independent t-test was run to determine whether there was a difference in the cost of sick leave (in USD) between female and male workers. The results showed that there is no statistically significant difference in the cost of sick leave between female workers (321.9 ± 48.9 USD) and male workers (294 ± 48.4 USD), t (154) = 0.3416, p = 0.7331 (based on a two-tailed significance level).

In the cases of the type of occupation and the type of medical care, where there are more than two groups, we first checked the normality of the distribution of sick days and their cost between the groups. Firstly, we used Levene’s robust test statistics for the equality of variances between groups, by days of sick leave and by their cost. Levene’s robust test statistic for days of sick leave per occupation category does not show homogeneity of variance (p = 0.0169). Similarly, Levene’s test for days of sick leave and type of medical care does not show homogeneity of variance between the groups (p = 0.00000001). When we performed the same test for the cost of the days of sick leave, we obtained the same result, with p = 0.0160 for types of occupation and p = 0.00000001 for type of medical care.

A Kruskal-Wallis H test was conducted to determine whether the number of days of sick leave was different for the five groups of workers: administrative technician (n = 25), director (n = 2), professional (n = 115), qualified manual worker (n = 10), and unqualified manual worker (n = 4). The test showed that there was no statistically significant difference in the number of days between the five groups (χ^^2^^_(2)_ = 6.255, p = 0.1809).

A Kruskal-Wallis H test was conducted to determine whether the number of days of sick leave was different for the five types of medical care: primary care (n = 2), infectious disease care (n = 75), chronic disease care (n = 52), maternity care (n = 13), and post-operative care (n = 7). The test showed that there was a statistically significant difference in the number of days between the five groups (χ^^2^^_(2)_ = 45.329, p = 0.0001).

A Kruskal-Wallis H test was conducted to determine whether the costs of sick leave were different for the five groups of workers: administrative technician (n = 25), director (n = 2), professional (n = 115), qualified manual worker (n = 10), and unqualified manual worker (n = 4). The test showed that there was a statistically significant difference in the costs between the five groups (χ^^2^^_(2)_ = 25.319, p = 0.0001).

A Kruskal-Wallis H test was conducted to determine whether the costs of sick leave was different for the five types of medical care: primary care (n = 2), infectious disease care (n = 75), chronic disease care (n = 52), maternity care (n = 13), and post-operative care (n = 7). The test showed that there was a statistically significant difference in the costs between the five groups (χ^^2^^_(2)_= 37.864, p = 0.0001).

## DISCUSSION

The main finding of this study is that there is no statistical difference between the number of days of sick leave taken by female and male workers, and no statistical difference in the cost of sick leave by gender. This refutes the idea that there are different trends and costs of absence depending on employee’s gender.^^26^,^27^^ Another main finding of this study is that the main cause of sick leave, and therefore of the cost of absence, is chronic disease. It confirms that employers can incur in cost due to sick leave for chronic diseases, not only for occupational diseases.^^28^^ Thus, it is important to design activities at work to prevent chronic illness. Another relevant finding of this study is that there is a difference between the mean number of days of sick leave according to the type of medical care. Also, there is a difference between the mean number of days of sick leave and of cost according to the type of medical care required and the type of occupation. This could be a huge problem, not only for companies, but also for the health system. If we consider the bias toward healthy workers and the view that chronic illness only impacts older people, this also becomes an opportunity to act in support of comprehensive workers’ health, not only in relation to occupational diseases but also in relation to chronic disease. This is one of the most important findings of this article for health and safety at work or for occupational health insurance companies. Among the limitations of this article is the short study period of one year and the number of sick leave registers.

However, we think that this study has some strengths that enable it to overcome previous limitations: the quality of the data is high because the database directly came from the company’s human resources department, and we had detailed information about medical diagnoses as recorded in the sick leave register. Additionally, we had information about daily wages, and the database was systematically completed by the company’s human resources department; thus, the estimation of the cost of sickness absence is accurate. We established the indirect cost of disease with better accuracy than the other studies previously cited, because the data gave us the daily wage; this is one of the most prominent strengths of this study, despite the size of the sample and the study period.

This is a strength of our study compared with the previous studies, such as those by MacGregor & Cunningham^^10^^ and von Vultée,^^15^^ which only used, for example, self-reported sickness absences.

We also find a mean of 5 days of absence for sickness, which is similar to the figures reported by MacGregor and Cunningham^^10^^ and Dale-Olsen^^16^^ in Finland. In our company, there was no performance pay, but all other aspects were equal to those of previous studies. It is remarkable that people with higher salaries tended to have fewer days of sick leave. We did not find any records of mental health problems in the sick leave records, contrary to what was found in the von Vultée^^15^^ study. The main cause of sick leave was MSDs, which have previously been claimed about the main cause of diseases in the working population age.

## CONCLUSIONS

This study demonstrates that the pattern of the sick leave that does not show statistical differences by gender related to days off work and cost. Also, we did not find statistical difference by occupation type related to the cause of the sick leave, but we found statistical difference by type of medical care. This is related to the importance of chronic disease as the main cause of absenteeism in this case; hence, this reinforces the need to incorporate programs or activities for promoting of health in such a way that workers suffer less from chronic diseases. Chronic diseases are those that generate the highest number of days of disability; therefore, organizations must be concerned with creating healthy work environments to avoid this cost for sick leave. MSDs was the most prevalent cause of sickness absence, which may be associated with the work, since the sample had administrative occupations and MSDs are the main burden of disease on working population age. Sickness absence is an issue that should the reason of concern to managers, since lost time generates significant cost for organizations. Understanding the causes associated with this absence will be important to generate actions that help mitigate its impact. Managers and occupational health specialists should understand the health condition of their employees to plan better health programs in the workplace.
